# Mechanical Behavior and Performance Degradation of Structural Cables in Buildings: A Comprehensive Review

**DOI:** 10.3390/ma18245502

**Published:** 2025-12-07

**Authors:** Xu Chen, Hai Zhang, Hongbo Liu, Jianshuo Wang, Yutong Zhang, Liulu Guo, Zhihua Chen, Marta Kosior-Kazberuk, Julita Krassowska

**Affiliations:** 1 School of Civil Engineering, Tianjin Chengjian University, Tianjin 300384, China; xchen_24@163.com (X.C.); zhanghai@tcu.edu.cn (H.Z.); zhchen@tju.edu.cn (Z.C.); 2 Joint Research Centre for Protective Infrastructure Technology and Environmental Green Bioprocess, Tianjin Chengjian University, Tianjin 300384, China; 3 School of Civil Engineering, Hebei University of Engineering, Handan 056038, China; hbliu@tju.edu.cn; 4 Tianjin Key Laboratory of Civil Structure Protection and Reinforcement, Tianjin Chengjian Universiy, Tianjin 300384, China; 5 School of Civil Engineering, Tianjin University, Tianjin 300072, China; zhangyt@tju.edu.cn (Y.Z.); guoliulu@163.com (L.G.); 6 Department of Building Structures and Structural Mechanics, Faculty of Civil Engineering and Environmental Sciences, Bialystok University of Technology, 15-351 Bialystok, Poland; j.krassowska@pb.edu.pl

**Keywords:** architectural structural cables, mechanical characteristics, stress relaxation and creep, high-temperature degradation, corrosion-induced degradation, post-fracture mechanical behavior

## Abstract

Owing to their lightness, high strength, flexibility, and design adaptability, cables have been extensively employed in architectural engineering. As cables are primary load-bearing components in long-span spatial structures, a profound understanding of their mechanical behavior is essential for structural design and safety evaluation. This paper presents a systematic review of the physical and mechanical properties of cables commonly used in building structures, offering reference data for key performance indicators. The mechanical responses and influencing factors pertaining to major types of cables—such as semi-parallel wire strand (SPWS), Galfan-coated steel strand (GSS), and full-locked coil wire rope (LCR)—are thoroughly examined. This review covers five critical aspects: fundamental cable characteristics, stress relaxation and creep, mechanical performance under high temperatures, corrosion-induced degradation, and post-fracture behavior after fatigue-induced wire breaks. It identifies key mechanical parameters, including elastic modulus, axial stiffness, bending stiffness, and the coefficient of thermal expansion. The degradation behavior of cables under high-temperature and corrosive conditions is examined, highlighting the superior corrosion resistance of LCR and GSS. Furthermore, the redistribution of stress and residual capacity after the rupturing of steel wires is elucidated. Based on recent studies, prospective directions are suggested to address current knowledge gaps and advance design strategies focused on durability and performance for forthcoming cable-supported structures.

## 1. Introduction

Structural cables are essential load-bearing elements in modern long-span buildings, including stadiums, exhibition halls, and airports. Their low weight, high strength, and excellent flexibility allow architects and engineers to create transparent and aesthetically striking spatial forms that are difficult to achieve with conventional steel or concrete members. Cable-supported systems efficiently transfer tensile forces, thereby reducing both self-weight and material consumption.

Unlike solid steel members, cables exhibit complex nonlinear mechanical behavior. This behavior stems from their helical geometry, inter-wire frictional contact, manufacturing-induced residual stresses, and varying boundary conditions [1]. These factors make accurate prediction of cable performance a persistent challenge in structural engineering, especially for long-term reliability assessment.

**Figure 1:**
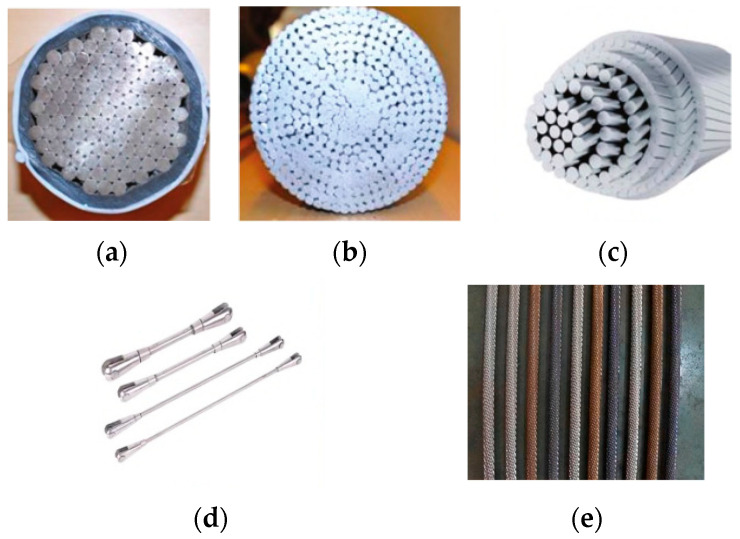
Common types of architectural cables: (**a**) SPWS, (**b**) GSS, (**c**) LCR, (**d**) steel rod, and (**e**) SSC [2,3].

The main types of structural cables used in architecture include semi-parallel wire strands, Galfan-coated steel strands, fully locked coil wire rope, steel rods, and stainless-steel cables (Figure 1). To ensure clarity and consistency throughout this review, the main types of structural cables discussed are uniformly classified and abbreviated as follows: semi-parallel wire strands (SPWSs), Galfan-coated spiral strands (GSSs), fully locked coil rope (LCR), stainless-steel cables (SSCs), and conventional spiral strands (SSs). Conventional spiral strands (SSs) are multi-layer helically wound steel wires, typically galvanized or uncoated. These abbreviations are used consistently throughout this manuscript.

SPWSs are manufactured by twisting parallel high-strength steel wires at a small lay angle (2–4°) into a dense bundle, which is then tightly bound and sheathed. Leveraging mature bridge cable technology, SPWSs applied in building structures often comply with established bridge industry standards. Typical tensile strength grades are 1670 MPa, 1770 MPa, 1860 MPa, and 1960 MPa [4]. Notable examples include the Century Lotus Sports Stadium and the Beijing University of Technology Badminton Gymnasium. GSSs consist of steel wires coated with a zinc–5% aluminum–rare-earth alloy. This coating provides superior corrosion resistance compared to conventional galvanization. Common strength grades are 1570 MPa, 1670 MPa, and 1770 MPa [5]. Examples of their application include the Tianjin University of Technology Gymnasium and Ya’an Tianquan Gymnasium. LCRs comprise inner layers of circular wires and outer layers of Z-shaped wires. This configuration creates a dense, sealed outer surface that effectively prevents moisture ingress and reduces internal wear, offering excellent corrosion resistance. The tensile strength grades range from 1470 MPa to 1960 MPa [6]. Representative projects include Changchun Olympic Park and the National Speed Skating Oval. Steel rods consist of a solid rod with end connectors. They are commonly used in suspendomes and cable domes and exhibit mechanical behavior similar to that of ordinary structural steel. SSCs provide excellent corrosion resistance and long-term durability. They are widely applied in prestressed and transparent facade structures, such as the Louvre Pyramid and Shenzhen Bay Sports Center [7]. Typical tensile strength grades are 1320 MPa, 1420 MPa, and 1520 MPa [8].

Numerous studies have investigated the behavior of structural cables under diverse mechanical and environmental conditions. However, the majority of these studies focus on one or two types of cables or isolated degradation mechanisms within laboratory settings. For example, Liu et al. [9] investigated the high-temperature mechanical properties and creep behavior of SSs. Lou et al. [10,11] examined prestress loss in twisted and parallel wire strands at elevated temperatures. Sun et al. [12,13] analyzed the thermo-mechanical responses of GSSs and SSCs. Yu et al. [14] explored the corrosion evolution and post-corrosion mechanical performance of SPWSs. Despite these contributions, systematic comparisons across different types of cables and investigations of coupled degradation mechanisms remain scarce.

Although standards such as JGJ257-2012 [15] and EN 1993-1-11:2006 [16] provide reference values for material properties, significant discrepancies persist in the reported elastic modulus values and coefficients of thermal expansion. These inconsistencies arise from differences in material composition, manufacturing processes, and the lack of standardized test protocols. The design and durability assessment of cable systems often rely on simplified assumptions that fail to reflect actual in-service conditions, introducing uncertainty into safety evaluations.

In recent years, research has increasingly focused on multi-physics coupling effects, including corrosion–fatigue interaction [17,18], thermo-mechanical degradation [19], and long-term creep-relaxation coupling [20]. At the same time, emerging technologies such as digital twins, structural health monitoring (SHM), and data-driven modeling are providing powerful tools that can improve prediction accuracy [21,22]. The integration of these modern approaches with traditional experimental and analytical frameworks remains a substantial challenge.

This paper presents a comprehensive review of the mechanical behavior and performance degradation of structural cables used in buildings. Unlike earlier reviews that focused mainly on bridge stay cables or examined individual degradation mechanisms in isolation, this study provides an integrated assessment tailored to architectural applications. This review synthesizes recent progress on material and mechanical properties, temperature- and environment-dependent behavior, corrosion evolution, fatigue performance, stress relaxation, and post-fracture response. Special emphasis is placed on large-diameter cables typically used in long-span roofs and façades, for which systematic experimental data and reliable design guidance are still scarce. By integrating findings from material-level tests, component-scale analyses, and practical engineering experience, this work clarifies the current state of knowledge, identifies critical methodological gaps, and outlines future research directions to advance the durability-oriented and performance-based design of next-generation cable-supported structures.

## 2. Systematic Review Methodology and Bibliometric Landscape

This section describes the comprehensive methodology used for this review. It details the systematic process followed to identify and analyze the relevant literature, ensuring we employed a reproducible and unbiased approach. A bibliometric analysis is then used to provide an overview of the research field, identifying critical trends, contributors, and focus areas.

### 2.1. Review Methodology

A systematic literature search was conducted in accordance with the PRISMA framework to ensure comprehensive coverage. Publications were retrieved from Web of Science (WoS) and China National Knowledge Infrastructure (CNKI), with supplementary full-text searches in ScienceDirect and SpringerLink.

The search combined Boolean operators (“AND” and “OR”) to capture two main dimensions: types of structural cables and their mechanical or degradation behaviors.

The representative search strings included

(“structural cable” OR “steel cable” OR “stainless steel cable” OR “semi-parallel wire strand” OR “Galfan-coated steel strand” OR “full-locked coil wire rope” OR “spiral strands”) AND (“characteristic” OR “elastic modulus” OR “axial stiffness” OR “bending stiffness” OR “coefficient of linear expansion” OR “thermal expansion” OR “stress relaxation” OR “creep” OR “mechanical properties” OR “elevated temperature” OR “corrosion” OR “wire breakage” OR “fretting fatigue”)

**Figure 2:**
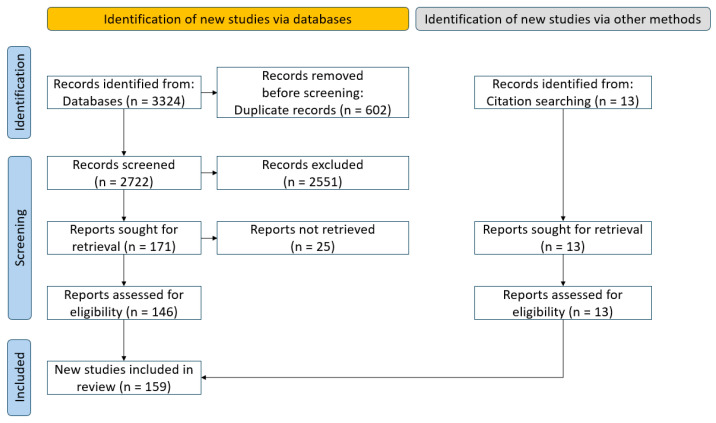
PRISMA flow diagram of the literature selection process.

Duplicate records were eliminated, and the remaining studies were screened based on titles, abstracts, and full texts according to standardized inclusion and exclusion criteria. Studies that concentrated on non-structural cables, parallel-wire strands, or the overall behavior of entire cable-supported structures were excluded throughout this process. Studies that concentrated solely on bridge cables were excluded because of substantial differences in structural scale and service conditions when compared to architectural cables, which are generally shorter, stockier, and exposed to unique boundary and environmental factors. The comprehensive screening process is depicted in Figure 2.

### 2.2. Bibliometric Analysis of the Research Landscape

This section presents a bibliometric analysis of the literature spanning the past 15 years. The objective is to characterize the current research landscape regarding structural cables in buildings. The analysis centers on two pivotal aspects: the temporal evolution of the annual publication volume and the statistics on high-frequency keywords. The results offer a macroscopic overview of research activity and dominant themes within the field. Furthermore, they lay the foundation for the detailed technical discussions presented in the subsequent sections.

#### 2.2.1. Publication Trends

**Figure 3:**
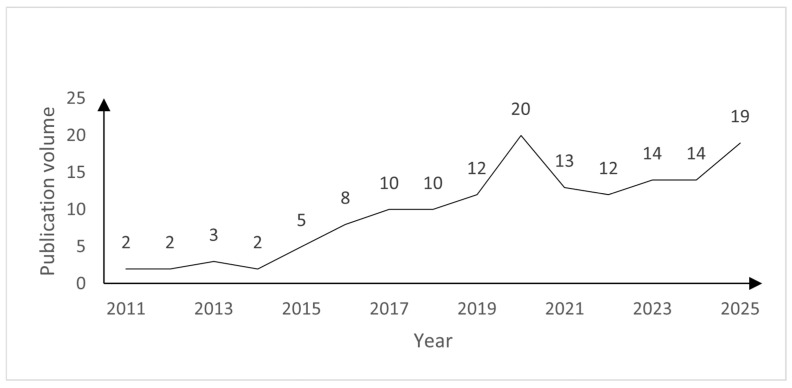
Annual publication volume regarding structural cables in buildings.

The temporal distribution of publications indicates a growing interest in cable performance research over the past 15 years (Figure 3). This surge aligns with modern architecture’s increasing adoption of cable-supported structures, which demand higher safety, durability, and performance under extreme conditions. The rise in publications also corresponds to advancements in material science, computational modeling, and experimental techniques, enabling more detailed investigations into cable behavior under thermal, mechanical, and environmental loads. The trend suggests that research is increasingly focusing on addressing the complex interplay between cable geometry, material properties, and environmental factors in order to address the need for more accurate predictive models and design guidelines.

#### 2.2.2. Keyword Statistical Analysis

The statistics of high-frequency keywords (Table 1) clearly reveal the dominant research themes and their interconnections. Terms related to fundamental mechanical behavior, such as “mechanical properties” and “cable”, remain central. Specific cable configurations, including “wire rope” and “spiral strand”, are frequently studied structural forms. The prominent degradation mechanisms are “creep”, “corrosion”, “relaxation”, “elevated temperature”, and “fretting fatigue”, reflecting a strong and growing emphasis on long-term durability and environmental resistance. From a methodological perspective, the frequent appearance of “finite element analysis” alongside experimental keywords highlights the prevailing dual reliance on numerical simulation and physical testing. This combination indicates a clear trend toward integrating multi-physics modeling with experimental validation to address complex mechanical responses and improve service-life prediction.

## 3. Fundamental Cable Characteristics

This section reviews the fundamental properties that define the mechanical behavior of structural cables in buildings. Characteristics such as elastic modulus, axial stiffness, bending stiffness, and the thermal expansion coefficient are discussed. These parameters are essential for predicting structural response under loads and environmental changes. Unlike monolithic steel elements, cables exhibit complex behavior due to their helical construction, inter-wire contact, and fabrication-induced stresses. Although design standards provide reference values, significant variations exist across cable types and loading conditions. This section summarizes current knowledge, highlights practical implications for structural design, and identifies research needs for improved modeling and performance prediction.

### 3.1. Elastic Modulus

When a cable experiences axial tension, the strain in individual wires is different from the overall axial strain acting on the cable [23]. As a result, the apparent elastic modulus of the cable is influenced not only by the intrinsic material properties of the constituent wires but also by geometric effects and inter-wire interactions. This effective modulus plays a crucial role in determining structural stiffness, prestress levels, and load redistribution in cable-supported systems.

According to the Chinese standard GB50017-2017, the elastic modulus for structural steel design is typically 2.06 × 10^5^ MPa without explicitly considering the helical geometry of cables [24]. The European standard EN 1993-1-11:2006 states that the modulus of steel cables varies with stress level, pretension, and loading history, and it recommends experimental determination [16]. The Chinese specification JGJ 257-2012 provides recommended elastic modulus values for different types of cables (Table 2) [15].

Among the various types of cables, SPWSs exhibit the highest effective elastic modulus due to their small lay angle. In contrast, steel wire ropes exhibit significantly lower stiffness because of their multiple helical layers and less compact arrangement. An increase in the number of helical layers leads to greater deformation under tension, which reduces the apparent elastic modulus. The composite nature of helically wound wires means cables experience not only axial force but also bending moment and torque under load, introducing additional stress states that result in an effective elastic modulus lower than that of the constituent wires. A cable’s elastic modulus is intrinsically linked to wire properties, which exhibit inherent uncertainties during manufacturing. Yu et al. [25] quantified the impact of uncertainties in wire properties on the global elastic responses of cables. They found that overall elastic modulus is predominantly determined by the wire’s intrinsic modulus, whereas the plastic tangent modulus is associated with the wire’s yield strength, ultimate strength, and ultimate strain.

### 3.2. Axial Stiffness

**Figure 4:**
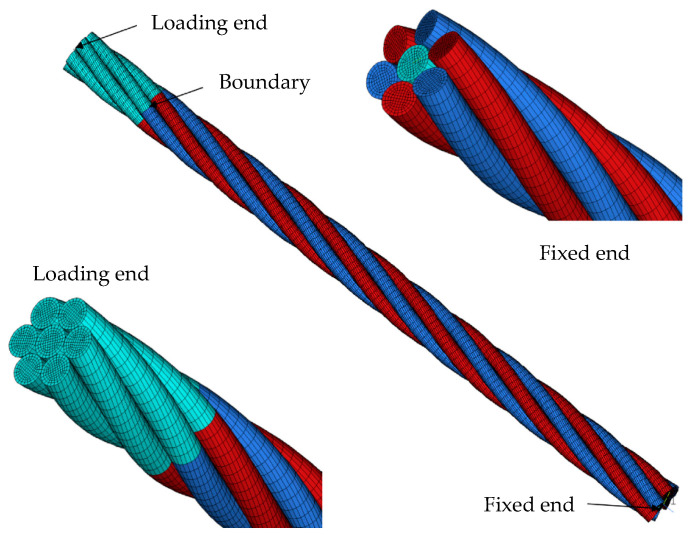
Spiral strand model incorporating a high-strength boundary section (Different colors indicate distinct element parts for contact modeling) [37].

A cable’s axial stiffness is influenced by various factors, including elastic modulus, strand configuration, and boundary conditions. Numerous analytical models have been developed to predict the axial tensile behavior of spiral strand cables [26,27,28,29,30,31,32,33]. These models are based on linear elastic assumptions and reasonable simplifications, providing approximate estimates of axial stiffness. However, they typically do not capture nonlinear deformation characteristics [34]. Lesnak et al. [35] introduced a refined wire rope model grounded in experimental data. This model incorporates the elastoplastic deformation of individual wires, allowing for accurate predictions of the tensile response and load-bearing capacity under axial loading. Hroncek et al. [36] proposed a simplified finite element model utilizing beam elements. This approach enables efficient predictions of load-carrying capacity, plastic strain distribution, and deformation behavior under tensile loading. Yu et al. [37] investigated the axial mechanical performance of SSs. They utilized finite element models that incorporated reinforced boundary sections to simulate realistic end constraints (Figure 4). The results indicated that frictional effects exert a negligible influence on axial stiffness under uniform tension.

Utting et al. [38,39] studied the axial–torsional behavior of SSs under different boundary conditions. They observed significant non-uniform load sharing among nominally identical wires, especially near free ends and close to the grips. Cables with fixed-end constraints displayed more-uniform stress distributions and higher overall stiffness. Judge et al. [40] developed an elastoplastic model for cables that accurately reproduced axial tensile behavior. The model included inter-wire friction but neglected manufacturing-induced residual stresses. Peng et al. [41] examined the effect of lay angle on the axial stiffness and inter-layer force distribution in SPWSs. They found that larger lay angles increase axial stiffness and promote more-uniform tensile stress distribution among the wires.

### 3.3. Bending Stiffness

In cable-supported structures, transverse loads induce lateral displacement of the cables. The bending stiffness of the cables determines their resistance to this displacement. While cables are typically regarded as having negligible bending stiffness due to their high slenderness, this resistance becomes considerable in large-diameter or relatively short cables [42,43,44]. In these instances, bending stiffness significantly affects the dynamic response and tension estimation derived from vibration frequencies, especially in higher vibration modes.

**Figure 5:**
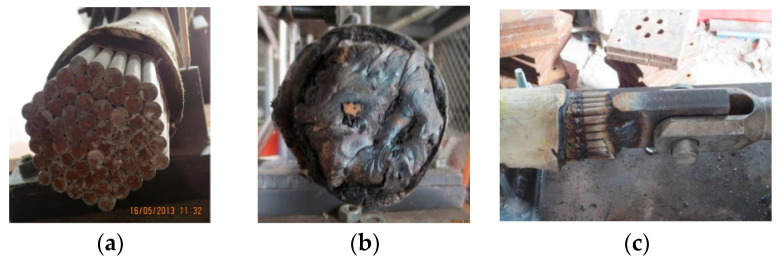
Cable end conditions tested: (**a**) free end; (**b**) welded end; and (**c**) end for pretension application [45].

Chen et al. [45] experimentally examined the effects of end conditions, pretension levels, and diameter on cable bending performance (Figure 5). They proposed simplified models for effective bending stiffness. Their results showed that GSSs maintained better structural integrity during bending than SPWSs or steel wire ropes. Bending stiffness increased with pretension but decreased with an increase in cable diameter.

Inter-wire frictional contact significantly influences bending resistance. Larger lay angles generally improve bending performance [46]. The theoretical minimum bending stiffness occurs when there is no inter-wire bonding, whereas the maximum is achieved under fully bonded conditions. Real cable behavior falls between these two extremes. Zhang et al. [47] demonstrated that neglecting wire-to-wire contact leads to underestimation of bending stiffness. Stiffness increases with the friction coefficient because friction provides additional resistance, even during steady sliding. Zheng et al. [48] examined the effects of helical angle and external sheathing. They concluded that inner layers experience higher static friction, while outer wires are more susceptible to slippage and fracture.

**Figure 6:**
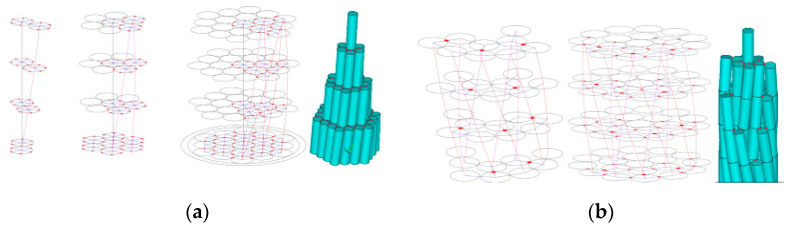
Finite element model of a cable incorporating inter-wire springs: (**a**) SPWS and (**b**) GSS (The red dots represent spring elements.) [49].

Numerical models have been extensively employed to simulate cable bending and frictional effects. Yu et al. [49] utilized tri-directional springs to model inter-wire compression and friction, effectively capturing sliding behavior in SPWSs and GSSs (Figure 6). Liang et al. [50] decomposed frictional forces into axial and tangential components to investigate the bending and dynamic response of SPWSs that include broken wires. Yang et al. [51] developed advanced finite element models to investigate variations in contact friction and axial wire slippage during bending. Bendalla et al. [52] analyzed stick–slip phenomena by incorporating residual interlock forces at contact points. They demonstrated that these forces significantly influence hysteresis and loading-history dependence. Sun et al. [53] simulated the frictional contact process induced by manufacturing. Their results indicated that the friction coefficient and self-rotation ratio have a strong impact on wire contact stress, residual strain, and plastic deformation. However, the combined effects of laying friction and installation-induced residual stress remain inadequately explored.

### 3.4. Coefficient of Thermal Expansion

Temperature fluctuations induce thermal expansion in cables, causing prestress relaxation and structural distortion [54]. The coefficient of thermal expansion (CTE) plays a crucial role in evaluating thermal–structural interactions in prestressed systems. Various standards specify distinct CTE values. For instance, ASCE 19-96 [55] suggests 1.15 × 10^−5^/°C, while EN 1993-1-11:2006 [16], GB 50017-2017 [24], and the Japanese code [56] advocate 1.2 × 10^−5^/°C, treating cables similarly to structural steel. Nonetheless, the helical configuration of cables results in effective CTE values that deviate from those of straight wires [57]. Drawing on extensive experimental evidence, CECS 212:2006 [58] offers the following standard values: 1.59 × 10^−5^/°C for wire ropes, 1.32 × 10^−5^/°C for SS, and 1.84 × 10^−5^/°C for steel tendons.

Lou et al. [10] proposed the following empirical formula for the CTE of high-strength steel wires at elevated temperatures:

(1) $$\alpha_{T}=1.34\times 10^{-8}T+9.77\times 10^{-6},\;\;\;\;T\leq 750\;{}^{\circ}C$$

where $\alpha_{T}$ is the CTE (/°C), and T is temperature (°C).

Zhou et al. [59] measured the free thermal expansion of steel strands between 100 °C and 800 °C. They developed the following empirical expression for the strand CTE:

(2) $$\alpha_{T}=3.74\times 10^{-9}\times T+1.22\times 10^{-5},\;\;\;\;20\;{}^{\circ}C\leq T\leq 800\;{}^{\circ}C$$

Here, $\alpha_{T}$ is the CTE (/°C), and T is the temperature (°C).

Sun et al. [3] investigated the thermal expansion of stainless-steel cables from 30 °C to 600 °C. They expressed the thermal expansion strain as Equation (3):

(3) $$\varepsilon_{T}=a\times {\left(T-30\right)}^{b},\;\;\;\;30\;{}^{\circ}C\leq T\leq 600\;{}^{\circ}C$$

where $\varepsilon_{T}$ is the thermal expansion strain, T is temperature (°C), and parameters a and b were obtained by fitting the experimental data. The instantaneous CTE can be derived from the first derivative of Equation (3).

**Figure 7:**
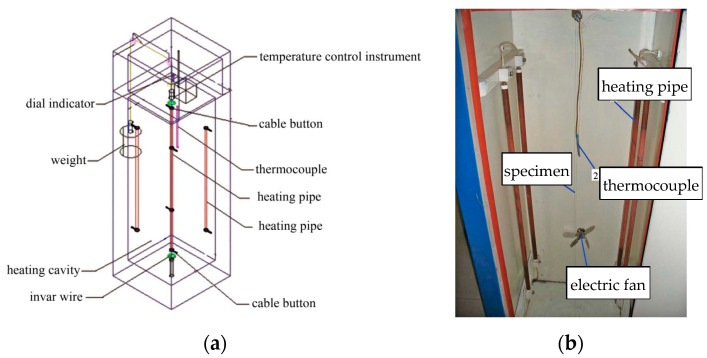
Air-heating apparatus: (**a**) schematic diagram, and (**b**) details of the physical instrument [61].

Chen et al. [60] measured the CTE of four types of cables using a water-bath thermal expansion tester. They found that steel wire ropes and SPWSs exhibit higher CTE values and are therefore more sensitive to temperature-induced prestress loss. Sun et al. [61] used an air-heating apparatus (Figure 7) to investigate the effects of wire diameter and lay length. Their results showed that the CTE decreases with an increase in lay pitch but increases with wire diameter. Numerical simulations by Sun et al. [62] revealed that, under thermo-mechanical coupling, the equivalent CTE increases with twist angle, wire diameter, and initial prestress, while it decreases with cable length.

The helical lay process introduces manufacturing-induced residual stresses that alter the effective axial thermal expansion behavior of cables. At elevated temperatures, the CTE increases. This effect, combined with high-temperature creep, leads to additional prestress loss and may compromise structural safety. Current experimental data are limited to specific cable specimens. Generalized prediction models applicable to untested types of cables remain unavailable.

Studies highlight a persistent gap between idealized theoretical models and the actual mechanical behavior of cables. Discrepancies in the elastic modulus CTE among different standards emphasize the significant influence of manufacturing-induced residual stresses, geometric configuration, and inter-wire friction. However, most numerical models currently depend on empirical parameter calibration, which limits their applicability to other types of cables. This gap between simplified theory and complex in-service conditions remains a major challenge for accurate performance prediction.

### 3.5. Comparison of International Standards

Design standards provide essential guidance for structural cables, yet significant differences exist among international codes regarding key mechanical parameters. These inconsistencies can lead to confusion and errors in engineering practice.

For the elastic modulus, the Chinese standard GB 50017-2017 [24] adopts a uniform value of 2.06 × 10^5^ MPa, typical of solid structural steel. This simplification may substantially overestimate the stiffness of stranded cables, especially wire ropes. In contrast, EN 1993-1-11:2006 [16] recognizes the complexity of cable behavior and states that the effective modulus depends on stress level, pretension, and loading history, recommending experimental verification. The Chinese cable-specific code JGJ 257-2012 [15] takes a more refined approach, providing type-specific values, e.g., 1.4 × 10^5^ MPa for single-strand wire ropes and 1.85–1.95 × 10^5^ MPa for SSs.

Similar discrepancies appear in regard to the CTE. ASCE 19-96 [55] recommends 1.15 × 10^−5^/°C, while EN 1993-1-11:2006 [16], GB 50017-2017 [24], and the Japanese code [56] adopt 1.2 × 10^−5^/°C, treating cables identically to solid steel. However, CECS 212:2006 [58] shows that helical geometry alters the effective CTE. Recommended values include 1.59 × 10^−5^/°C for wire ropes and 1.32 × 10^−5^/°C for SSs.

The primary risk stems from applying parameters derived from solid steel to stranded cables. Utilizing the elastic modulus of solid steel for wire ropes can lead to a significant overestimation of axial stiffness, which in turn results in inaccurate predictions of natural frequencies, force distribution, and deformation. Likewise, employing a uniform CTE for steel may either lead to underestimation of thermal effects on wire ropes or overestimation of these effects on steel strands, thereby impacting calculations related to prestress loss and temperature-induced responses.

These discrepancies underscore a critical issue: the behavior of cables is influenced not only by material properties but also by geometry and inter-wire interactions. Therefore, transitioning from prescriptive, simplified code values to performance-based design is imperative. For critical structures, reliance on product-specific test data, advanced numerical models accounting for composite behavior, or in situ monitoring is recommended. These approaches ensure a precise and dependable design, thereby accurately reflecting actual in-service performance.

## 4. Stress Relaxation and Creep

Stress relaxation and creep are two fundamental time-dependent deformation phenomena that significantly influence the long-term performance of prestressed and tensioned cable systems. Stress relaxation is a gradual decrease in stress under constant strain, while creep is a progressive increase in strain under constant stress. Both phenomena arise from viscoelastic or viscoplastic mechanisms. Their effects are notably affected by cable geometry, material composition, environmental conditions, and sustained stress levels. Under conditions of prolonged high stress, these effects can lead to force reduction, permanent deformation, and stiffness degradation [63]. As a result, they have implications for structural safety and serviceability. Therefore, accurate modeling of these effects is essential for reliably predicting durability and prestress retention in cable-supported structures.

### 4.1. Stress Relaxation

**Figure 8:**
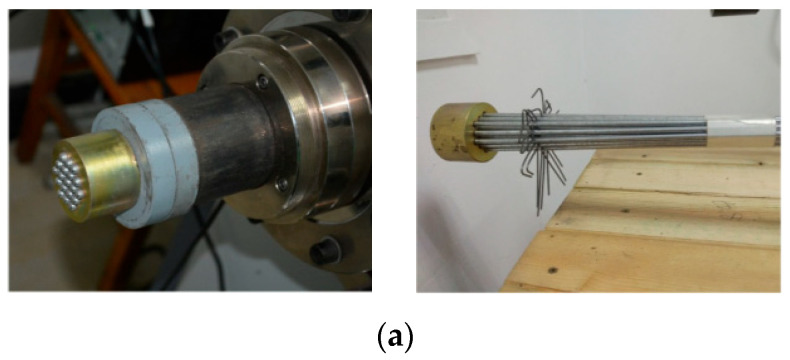

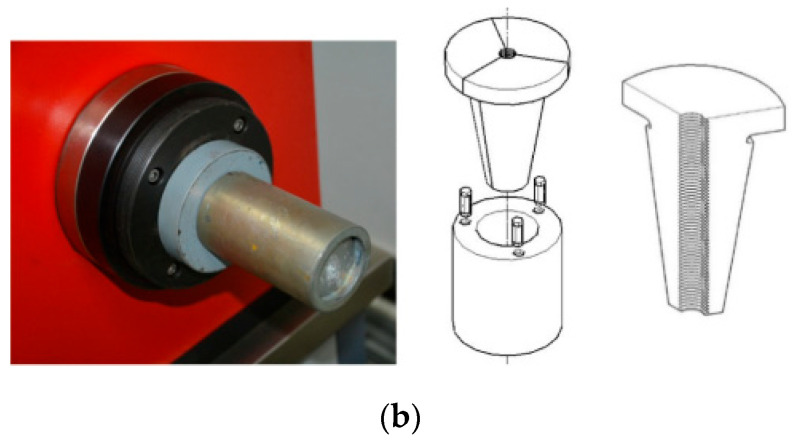
Cable anchorage configurations for relaxation testing: (**a**) SPWS and (**b**) GSS [64].

Wang et al. [64] conducted long-term relaxation experiments on SPWSs and GSSs. To minimize end slip and secondary effects, they utilized specialized anchorage systems (Figure 8). The results indicated a positive correlation between the relaxation rate, initial stress, and temperature. Based on these findings, the cited authors proposed empirical models for predicting long-term relaxation and provided recommended values for 50-year relaxation ratios. Notably, GSS cables exhibited a significantly higher relaxation rate than other types of cables. Their 50-year relaxation rate reached 24.727%, while the rates for other types of cables typically ranged from 2.5% to 4%.

Sun et al. [65] investigated the effects of initial prestress and diameter on the stress relaxation of GSSs and SSCs. Their findings indicated that the relaxation rate of GSS cables increases with both prestress and diameter. In contrast, the relaxation rate of SSCs remained independent of initial load and diameter, with an estimated 50-year value of approximately 8%.

Feng et al. [66] proposed a three-parameter viscoelastic constitutive model. They subsequently extended this framework into a multi-element viscoelastic model based on extensive long-term experimental data. This advanced model effectively captures the long-term relaxation behavior of steel cables. Atienza et al. [67,68] demonstrated that elevated manufacturing-induced residual stresses result in increased relaxation rates. Chen et al. [69] found that relaxation rates rise with larger lay angles, especially in the outer wire layers. Chen et al. [70] examined the combined effects of tension and bending. Their findings revealed that relaxation facilitates a more uniform contact pressure among wires, which in turn diminishes peak stress, frictional wear, and the risk of fatigue.

Numerical predictions of stress relaxation have been validated against long-term experimental tests. The models accurately replicate the decay pattern observed during the initial stage. However, quantitative discrepancies arise over extended periods, suggesting that temperature-dependent relaxation coefficients necessitate further calibration through prolonged testing. Stress relaxation, despite its localized benefits in reducing peak contact pressures and enhancing stress uniformity under combined loading conditions, is generally considered an adverse degradation mechanism. It leads to a decline in prestress, diminished axial stiffness, and progressive deformation in cable-supported structures. Although it offers some alleviation in specific scenarios, the overall impact of stress relaxation on structural design remains unfavorable.

### 4.2. Creep Behavior and Modeling

**Figure 9:**
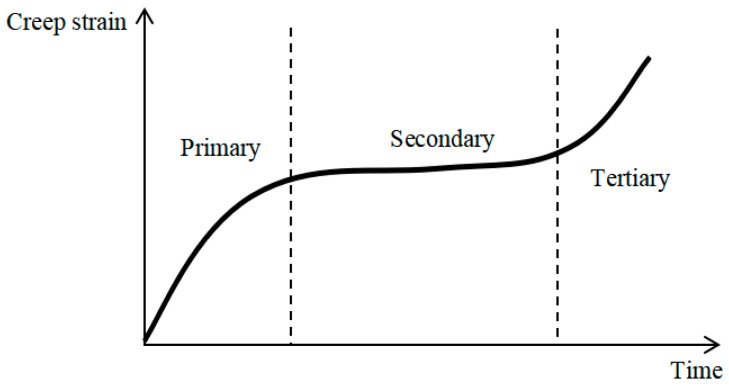
Characteristic stages of the creep curve.

Creep in cables is characterized by a gradual increase in strain under sustained tension. The typical creep curve consists of three distinct stages (Figure 9): the primary (transient), secondary (steady-state), and tertiary (accelerating) phases. At service-level stresses, cables primarily exhibit the first two stages. Tertiary creep occurs only under elevated temperatures or at stress levels nearing the ultimate strength.

Ivanco et al. [71] examined the effects of wire clearance and friction on the creep strain of SSs. Their study demonstrated that inter-wire clearance significantly increases creep strain. Zhang [72] developed a creep constitutive equation for steel cables and applied it to time-dependent analysis of cable-net structures. Liu et al. [9] and Du et al. [73] performed high-temperature creep tests on steel strands and proposed the following creep models:

(4) $$\varepsilon_{cr}=\frac{A}{m+1}\sigma^{n}t^{m+1}$$

where $\varepsilon_{cr}$ is creep strain, σ is equivalent stress, and t is total time. A, m, and n are creep parameters, fitted using test results and MATLAB based on a genetic algorithm [9].

(5) $$\varepsilon_{creep}=\frac{c_{1}}{c_{3}+1}\sigma^{c_{2}}t^{c_{3}+1}e^{-\frac{c_{4}}{T}}+c_{5}\sigma^{c_{6}}te^{-\frac{c_{7}}{T}}$$

where $\varepsilon_{creep}$ is creep strain, σ is equivalent stress, t is fire duration, T is temperature, and c_1_ to c_7_ are fitting parameters [73].

Sun et al. [60,74] measured high-temperature creep deformation in GSSs and SSCs and established corresponding models. They applied the Fields–Fields creep model to describe deformation at constant temperatures:

(6) $$\varepsilon_{cr}=at^{b}\sigma^{c}$$

where $\varepsilon_{cr}$ is creep strain; σ is stress; and t is time. The coefficients a, b, and c are parameters related to temperature.

Du et al. [75] investigated the high-temperature creep behavior of LCRs and GSSs. They reported that creep strain increases with both stress ratio and temperature. Zhang et al. [76] established a predictive framework utilizing a physics-enhanced multi-fidelity neural ordinary differential equation (NODE) model. This hybrid data-driven methodology demonstrated high accuracy in extrapolating long-term creep behavior from a limited set of experimental data.

Despite considerable advancements in our understanding of stress relaxation and creep, accurately predicting the long-term performance of cables remains a formidable challenge. This challenge stems from the intricate interplay among material viscoelasticity, cable geometry, and environmental influences. Most current empirical models are calibrated under accelerated, constant conditions, such as fixed temperatures and loads. These models fail to account for cumulative deformation and ratcheting effects that arise from cyclic thermal and mechanical loading in actual structures. As a result, their reliability for extrapolation over extended periods is constrained.

## 5. High-Temperature Mechanical Properties

As metallic tension components, steel cables inherently exhibit poor fire resistance. Exposure to elevated temperatures during fire events causes a sharp deterioration in their strength, stiffness, and ductility. This degradation can potentially trigger progressive structural collapse. Even if the global structure survives a fire, residual deformation and loss of material properties can critically compromise its post-fire load-carrying capacity. Therefore, a comprehensive understanding of mechanical performance both during fire exposure and after cooling is imperative. This knowledge is essential for the fire-resistant design, safety assessment, and reuse of prestressed and cable-supported structures.

### 5.1. Experimental and Numerical Investigations

Numerous studies have examined the mechanical degradation of cables at high temperatures and after cooling. These investigations provide essential experimental data and constitutive models for performance-based fire design. Representative experimental research is summarized in Table 3.

Liu et al. [9] investigated the influence of strand twisting, heating duration, and cooling methods on the tensile behavior of steel strands at elevated temperatures. Their results indicated a marked reduction in ultimate strength when temperatures exceeded 400 °C. This decline was accompanied by a loss of elastic stiffness. Liu et al. [79] examined the effect of water-quenching temperature on post-fire mechanical recovery. They proposed a piecewise relationship between cooling temperature and residual tensile strength, as presented in Equation (7). The steel strands exhibited brittle characteristics when cooled with low-temperature water. Conversely, they retained a degree of ductility when cooled with high-temperature water.

(7) $$f_{u,wt}=\left\{\begin{array}{c} 0.5259T+14.965,\;\;\;\;\;\;\;\;\;\;\;\;\;\;\;\;\;\;\;\;\;\;\;\;\;\;\;\;\;\;\;\;\;\;\;\;\;\;\;\;\;\;\;\;\;\;\;\;\;\;10\;{}^{\circ}C\leq T\leq 50\;{}^{\circ}C\\ -12457.56948e^{\left(-\frac{T}{10.38289}\right)}+141.12975,\;\;\;\;\;\;\;\;\;50\;{}^{\circ}C\leq T\leq 80\;{}^{\circ}C \end{array}\right.$$

Here, $f_{u,wt}$ is ultimate strength, and T is water-cooling temperature.

**Figure 10:**
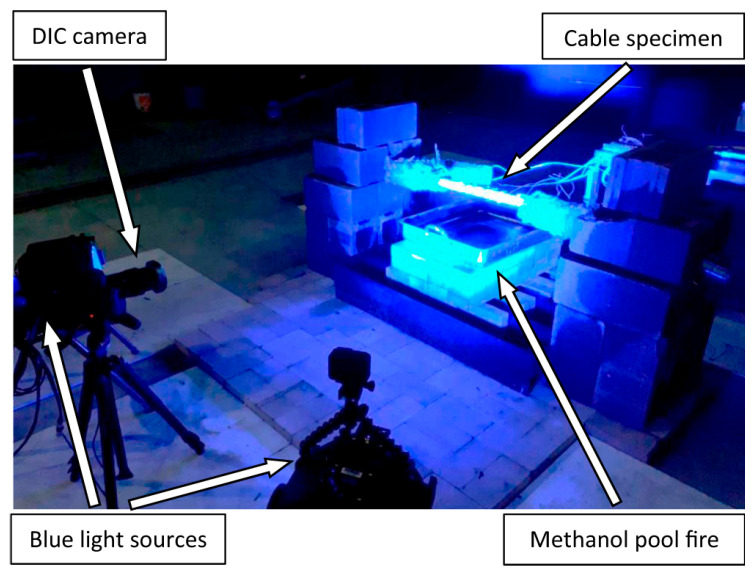
Typical digital image correlation and narrow-spectrum illumination test configuration [85].

Nicoletta et al. [85] experimentally studied the thermal responses of SSs and LCR under pool fire exposure (Figure 10). Their findings highlighted the influence of diameter on temperature distribution and thermal gradients. Watson et al. [86] developed finite element thermal models to predict transient temperature distributions in pool fire scenarios. Fontanari et al. [84] elucidated the load-transfer mechanisms and failure modes of LCR under severe thermal transients. Qu et al. [87] simulated the thermo-mechanical behavior of SSs and LCR in large-volume building fires. Du et al. [88] investigated the evolution of transient tension during localized fires. Their results showed that a rapid temperature rise causes immediate prestress loss, which can trigger local instability before material failure occurs.

Comparisons between numerical simulations and pool-fire tests show good agreement regarding overall temperature–time histories. However, simulations typically underestimate transient temperature gradients and residual deformation. These discrepancies highlight the need for improved boundary conditions and more accurate temperature-dependent material properties.

### 5.2. Comparison of the Performance of Different Types of Cables

SSCs exhibit superior performance at elevated temperatures, which is attributed to their stable austenitic structures. They retain approximately 50–60% of their room-temperature strength at temperatures exceeding 600 °C. However, their expensiveness restricts their widespread application. GSS cables offer excellent corrosion protection under normal conditions. However, the coating loses effectiveness above 400 °C as the zinc–aluminum layer deteriorates, accelerating the oxidation of the underlying steel.

The dense interlocking structure of LCR significantly modifies the internal mechanical and thermal transfer characteristics of the cable. This structure increases solid contact pathways and reduces internal voids. The practical implications of these structural features under transient, non-uniform fire exposure require further verification. The selection of an appropriate type of cable requires a balanced consideration of thermal resistance, cost, and post-fire performance.

Most experimental studies examining the mechanical properties of structural cables at elevated temperatures and in post-fire conditions have been performed under steady-state heating conditions. In these tests, a slow heating rate of approximately 10 °C/min is generally used. This testing protocol contrasts significantly with the transient temperature–time curves encountered in actual fire scenarios, which often display rapid heating rates that exceed 100 °C/min. The common steady-state heating approach fails to accurately capture the rapid thermal transients and temperature gradients characteristic of actual fires. This limitation results in calibrated models that may underestimate the rate and extent of degradation in stiffness and strength. The absence of standardized post-fire evaluation criteria hinders the accurate assessment of structural safety following a fire event.

## 6. Corrosion and Fretting Degradation Behavior

During long-term service, structural cables are inevitably subjected to corrosive environments and cyclic loading from wind, traffic, and thermal variations. The interaction between corrosion and fatigue is frequently compounded by fretting fatigue. This phenomenon arises from microscopic relative movements between adjacent contacting wires. Together, these mechanisms significantly accelerate microcrack initiation and wire breakage, constituting one of the most critical failure modes in cable-supported structures. Fretting markedly reduces fatigue life by promoting surface damage and compromising protective coatings. These mechanisms lead to wire breakage, which alters load distribution and compromises structural reliability. This section reviews degradation mechanisms induced by corrosion, fretting fatigue, and the mechanical performance following wire fracture.

### 6.1. Corrosion-Induced Degradation

**Figure 11:**
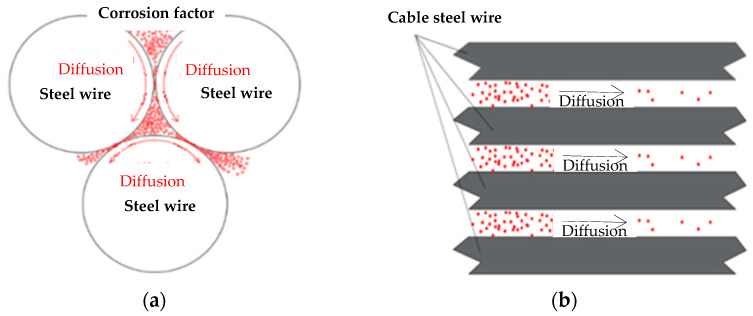
Transfer forms of corrosion factors in the cable: (**a**) radial transmission and (**b**) circular and axial transmission [90].

Guo et al. [89] developed a corrosion kinetics model for galvanized steel wires to simulate corrosion propagation in multi-wire cables with localized coating damage. Li et al. [90] proposed a spatial diffusion model for corrosive agents (Figure 11) based on an optimized back-propagation neural network. This model accurately captures the heterogeneous penetration process within cable assemblies. Yu et al. [91] proposed a semi-empirical stress corrosion model that correlates the corrosion rate with the applied tensile stress. Their simulations demonstrated that under mechano-electrochemical coupling, higher stress accelerates the growth of corrosion pits. This process reduces the residual strength of the material.

Yao et al. [92] experimentally studied the combined effects of corrosion and cyclic loading on steel strands. They proposed a corrosion–fatigue life prediction formula as a function of pit depth and stress amplitude (Equation (8)). Their results confirmed that tensile strength and elongation decrease markedly with an increasing corrosion depth, while higher stress amplitudes accelerate corrosion progression and intensify material degradation.

(8) $$N=10^{7}{\left(∆\sigma_{eqv}^{2/3}-{\left(185\right)}^{2/3}\right)}^{-2}$$

Here, N is corrosion fatigue life, and ${∆\sigma }_{eqv}$ is equivalent stress.

**Figure 12:**
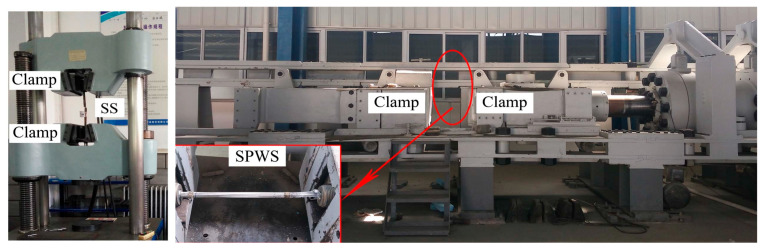
Tensile testing machine [93].

Chen et al. [93] performed tensile tests on corroded SSs and SPWSs (Figure 12) and proposed a generalized three-parameter corrosion model, as shown in Equation (9). They established an equivalent conversion relationship between atmospheric exposure and accelerated corrosion tests. This relationship enables the estimation of the recommended 50-year corrosion depth for cables with varying structural configurations. Their study revealed that galvanized coatings exhibit inferior corrosion resistance relative to protective paint coatings. The twisting of strands was found to enhance long-term resistance to rust.

(9) $$d_{u}\left(t\right)=A\times {\left(t+C\right)}^{B}$$

where $d_{u}\left(t\right)$ is the uniform corrosion depth of the galvanized steel wire in an accelerating corrosion test, A is the initial corrosion rate, B is rust trend, and C is the error correction factor.

**Figure 13:**
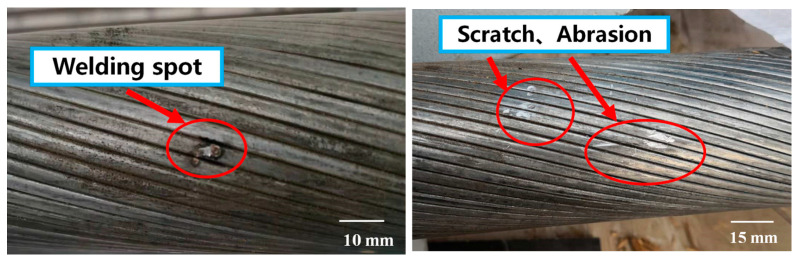
Damage modes of alloy coatings [95].

Zhang et al. [94] evaluated the corrosion performance of Galfan-coated LCRs in a natatorium environment and established a corrosion-rate model for Galfan coatings exposed to high humidity and chloride aerosols. Fang et al. [95] analyzed the mechanical behavior of Galfan-coated LCRs with intentional coating defects (Figure 13) after corrosion. Their study revealed that defective specimens were more susceptible to tensile failure than standard specimens with intact alloy coatings. Among the various types of defects, weld-point imperfections exhibited the most pronounced adverse effects.

Yu et al. [14] studied corrosion evolution paths and mechanical degradation in SPWSs under three sheath damage scenarios. The corrosive solution primarily infiltrated the cable through narrow gaps between the HDPE sheath and steel wires, with the diffusion direction governed by the cross-sectional arrangement of the wires. A denser wire configuration effectively impeded the penetration of corrosive agents into the inner layers.

Jikal et al. [96] investigated the influence of corrosion on the mechanical behavior of wire rope strands through accelerated corrosion and tensile tests. They introduced a correction factor to improve the Faupel theoretical model, enabling accurate prediction of residual load-bearing capacity and damage evolution at different corrosion levels. Their results showed that ultimate load-bearing capacity decreases markedly with corrosion duration, confirming a strong positive correlation between corrosion time and strength degradation. The corrosion-induced damage process can be divided into three distinct stages: initiation, stable propagation, and accelerated instability.

Tijani et al. [97] examined the effects of corrosion on the stiffness and ultimate strength of steel wire ropes and proposed a predictive model for evaluating the residual load-bearing capacity based on measurements of the residual rope diameter, as shown in Equation (10):

(10) $$F_{ur}=\left[D_{r}^{2}-\alpha_{y}\left(1-D_{r}^{2}\right)\right]F_{u}$$

where $F_{ur}$ is the residual force of the corroded wire, $F_{u}$ is the ultimate force of the undamaged wire, $D_{r}$ is the residual diameter ratio, and $\alpha_{y}$ is a coefficient of the mean value.

Guo et al. [98] investigated the surface morphology and corrosion behavior of LCRs and Z-shaped wires after accelerated corrosion tests. They established a corrosion kinetics model and a probability density function for surface corrosion depth. Based on these, they proposed a method for evaluating the residual mechanical performance of corroded LCRs. Their results showed that corrosion significantly reduces cable ductility, resulting in lower ultimate and fracture strain. The Galfan coating was found to effectively delay corrosion progression.

Subsequent studies confirmed that corrosion not only degrades surface morphology but also induces pitting and localized section loss. These defects cause stress concentration, micro-crack initiation, and eventual fracture, resulting in significant reductions in tensile strength and ductility. To quantify this degradation, numerical and analytical models that incorporate corrosion-depth distribution, pit evolution, and material-property deterioration within finite element frameworks have been developed. These models accurately capture the nonuniform stress fields and stiffness reduction induced by corrosion, enabling reliable prediction of residual strength and remaining service life.

Experimental and modeling results consistently indicate that corrosion-induced geometric and mechanical non-uniformity significantly influences the long-term performance of cable structures. This finding highlights the necessity of incorporating corrosion–mechanical coupling into durability assessments and design processes. Current corrosion-damage models align well with observations from electrochemical and salt-spray tests, accurately reflecting the nonlinear reduction in cross-sectional area and tensile strength over time. However, precise prediction of localized pitting evolution still requires detailed micro-structural calibration.

### 6.2. Fretting-Fatigue Behavior

Structural cables are frequently subjected to vibrations induced by wind, such as vortex-induced vibration and rain–wind-induced vibration. These dynamic actions generate lateral motion. Consequently, significant bending stresses concentrate at the connection and anchorage zones. These cyclic actions induce fretting fatigue, arising from small-amplitude relative slips between adjacent wires or between wires and anchorage components. This phenomenon produces localized contact stress and surface damage, which accelerate crack initiation and shorten service life.

Xu et al. [99] investigated spiral contact steel wires under the influence of the coupling of tension and torsion. They found that increasing the torsional angle intensified adhesion and wear. This condition reduced fatigue life through ductile fracture accompanied by secondary cracks. Huang et al. [100] reported that torsional deformation reduced breaking force by 2.6% and bending fatigue performance by 36%. Furthermore, they observed a shift in wear mechanisms from abrasive to surface fatigue. Peng et al. [101] highlighted that lubrication and a reduced twist angle effectively lowered the coefficient of friction and wear depth in multi-wire contact.

Huang et al. [102] investigated fretting wear in corrosive environments and found that acidic and seawater conditions exacerbated surface fatigue, thereby accelerating the degradation of tensile strength. Liu et al. [103] corroborated this synergy through experimental validation, demonstrating that corrosion intensified fretting scars beyond the cumulative effects of individual corrosion and wear damage. These findings collectively underscore the synergistic interaction between corrosion and fretting, which accelerates degradation in service cables.

From a modeling standpoint, Ahmad et al. [104,105] developed a damage-based finite element model that incorporated the degradation of Young’s modulus. This model effectively predicted the onset of fretting damage in both two-wire and strand configurations. Ahmad et al. [106] identified micro-slip zones of SSs that corresponded with experimental crack nucleation sites. Hu et al. [107] proposed an energy-based wear model that correlated wear depth with dissipated friction energy. Wang et al. [108] further extended this simulation to encompass the interplay between corrosion and wear, achieving accurate life predictions with a deviation of less than 1%.

Experimental and numerical investigations consistently indicate that fretting-fatigue damage initiates in regions of partial slip, which are characterized by elevated tangential stress. This damage evolves under the combined effects of torsion, contact geometry, and corrosion. Experimental findings illustrate a transition from abrasive mechanisms to surface fatigue mechanisms. Simultaneously, simulations validate the associated trends in stress localization and modulus degradation.

### 6.3. Post-Fracture Mechanical Behavior

Recovery length, defined as the distance from the fractured end over which a broken wire regains its intended load share, is a critical indicator for evaluating the damage tolerance and residual strength of a cable. Geometric parameters such as wire diameter, lay angle, and the number of layers significantly influence this recovery length. Raoof [109] investigated the effects of axial load and external pressure on the recovery length of broken wires in multi-layer SSs, finding that recovery length increases with an increase in axial load but decreases as external pressure increases. Peng et al. [110,111,112,113] derived symmetric and asymmetric fracture mechanics models for SPWSs under static tension, analyzing the post-breakage tension distribution in wires. Within the recovery length, the tensile force is non-uniformly distributed among wires at the same cross-section, with this non-uniformity being most pronounced at the location of the fracture. Beyond this length, the tension distribution across wire layers becomes essentially uniform. In cases of asymmetric breakage, the tensile forces acting on wires at the fracture site are significantly greater on the side of the break than on the opposite side. Wang et al. [114] studied the influence of sheath gripping force and wire helical angle in SPWSs on broken wire recovery length, discovering that the recovery length decreases when the broken wire is located in an inner layer. A stronger sheath gripping force also results in a smaller recovery length. Sun et al. [115] simulated the processes of corrosion and fracture in SPWSs via FE analysis to study the effect of corroded wire fractures on the cables’ mechanical properties. Their findings indicate that when wire corrosion occurs, the recovery length of the fractured wire increases significantly. Furthermore, the recovery length also increases as the percentage of broken wires increases.

Recovery length is highly sensitive to cable geometry, boundary constraints, corrosion state, and the number and configuration of broken wires. Current models show that corrosion and multiple fractures can extend the disturbed zone by several times relative to intact conditions, leading to greater non-uniform stress distribution and accelerated loss of residual strength. Accurate quantification of recovery length under coupled corrosion–fatigue conditions is essential for reliable remaining-life prediction and safe continued use of damaged architectural cables.

## 7. Discussion and Synthesis

Existing research has substantially deepened our understanding of structural cable behavior. Nonlinear stiffness characteristics have been firmly linked to helical geometry, inter-wire friction, and manufacturing-induced residual stresses. Time-dependent studies have consistently shown that stress relaxation and creep cause prestress losses of 8–25% over 50 years, with GSSs exhibiting the highest rates. High-temperature tests have revealed that steel strands lose more than 50% of their strength at above 400 °C and that steady-state heating protocols significantly underestimate transient stiffness degradation observed in real fire scenarios. Fretting-fatigue and fretting-wear studies consistently show that micro-scale wire movements rapidly initiate surface cracks, with corrosion accelerating damage by more than an order of magnitude. Corrosion–fatigue studies have further confirmed that pitting and fretting scars act as preferred crack-initiation sites, leading to sudden wire rupture long before uniform section loss would predict failure.

These specific findings expose several critical limitations that prevent reliable long-term performance prediction. First, while steady-state tensile tests at slow heating rates (10 °C/min) provide essential material properties under controlled conditions, they fail to replicate rapid heating rates and severe internal temperature gradients. Consequently, thermo-mechanical models calibrated from these slow-rate tests often underestimate transient temperature gradients and instantaneous prestress loss. Second, corrosion research continues to rely on accelerated salt-spray or uniform exposure protocols, whereas natural atmospheric corrosion under cyclic humidity–temperature conditions produces markedly different localized pitting morphologies that current kinetics models fail to capture. Third, relaxation and creep data obtained under constant loads and at constant temperatures cannot explain the accelerated prestress loss observed in field-monitored cables subjected to daily and seasonal fluctuations. Fourth, although fretting fatigue is now recognized as a dominant wire-breakage mechanism, most finite element models still treat inter-wire contact as perfectly bonded or purely frictional without incorporating progressive wear and modulus degradation, leading to predicted fatigue lives that can exceed experimental values.

Moreover, the scarcity of long-term field data means that the synergistic evolution of corrosion, fretting, relaxation, and thermal degradation remains largely unquantified in real structures. Advanced numerical frameworks, despite accurately reproducing isolated phenomena at the wire or strand level, continue to idealize boundary conditions and initial imperfections (e.g., pretension scatter, manufacturing residual stresses, and sheath-gripping variability). Consequently, simulated global responses of large-span cable-supported systems still deviate significantly from measured structural behavior.

These gaps explain why current models remain inadequate for life-cycle design and post-event assessment of architectural cables. Addressing them requires a decisive shift toward transient, multi-physics experiments; long-term field monitoring; and fully coupled numerical platforms that integrate manufacturing-to-degradation processes from the outset. Only through these advancements can the durability-oriented, performance-based design of next-generation cable-supported structures be achieved.

## 8. Outlook and Research Roadmap

**Figure 14:**
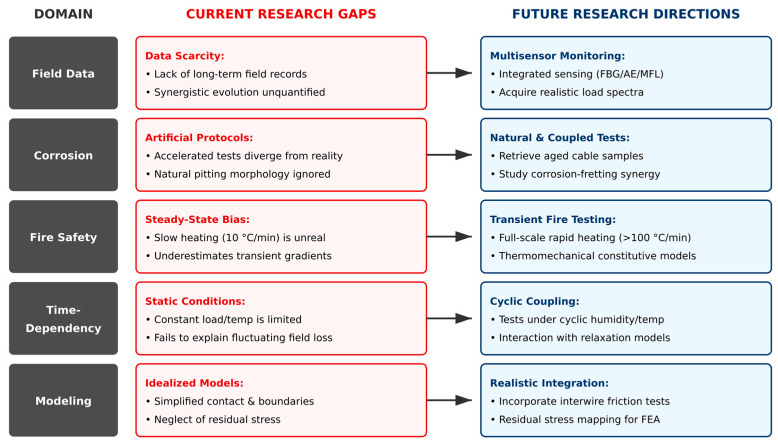
An overview of major research gaps and future directions in architectural cable studies.

To address the core challenges discussed in Section 7 and advance the field toward performance-based design and intelligent maintenance, we propose the following key research directions (Figure 14):

1. Implement multi-sensor long-term field monitoring for in-service architectural cables. Install integrated FBG, acoustic emission, and magnetic flux leakage sensors on a minimum of three long-span roofs. Monitor tension, wire breakage, corrosion rates, and fretting damage continuously. Acquire realistic multi-physics load spectra and degradation trajectories under actual service conditions.
2. Combine long-term natural corrosion monitoring with fretting-fatigue testing. Retrieve cable samples from in-service cable-supported structures with extensive operational histories. Perform detailed metallographic analysis of pitting and fretting scars, alongside residual strength and fatigue tests. Establish quantitative models for the synergy between corrosion and fretting. Determine reliable equivalence relationships between natural exposure and accelerated laboratory tests.
3. Conduct full-scale transient fire tests on large-diameter cables with heating rates exceeding 100 °C/min. Record radial and axial temperature distributions, thermal torsion, real-time prestress evolution, and post-fire residual mechanical properties simultaneously. Develop validated thermomechanical constitutive relationships for nonuniform rapid heating conditions. Establish unified post-fire assessment criteria to ensure the safe reuse of cable-supported structures.
4. Conduct coupled experiments on corrosion and relaxation. Perform prolonged relaxation tests on pre-corroded strands subjected to cyclic variations in temperature and humidity. Quantify the impact of localized corrosion on the acceleration of stress relaxation and the alteration of long-term stiffness degradation. Integrate these interactions into prediction models for life cycle performance.
5. Conduct inter-wire friction tests and residual stress measurements on full-scale cables. Obtain realistic coefficients of friction and representative initial residual stress fields for typical types of cables. Integrate these experimentally derived parameters into refined finite element models to significantly enhance the predictive accuracy of axial and bending stiffness and the recovery length of fractured wires.

The integration of these efforts will enhance the safety, durability, and intelligence of next-generation cable-supported structures, ensuring their high performance under increasingly complex and demanding conditions.

## Figures and Tables

**Table 1:** Statistics of high-frequency keywords.

Number	Keyword	Frequency	Number	Keyword	Frequency
1	Cable	26	16	Wire rope strand	8
2	Mechanical properties	25	17	Model	7
3	Wire rope	17	18	Mechanics	7
4	Steel wire	13	19	Tension	7
5	Steel wire rope	13	20	Engineering mechanical	6
6	Spiral strand	12	21	Locked coil wire rope	6
7	Creep	12	22	Residual strength	5
8	Corrosion	11	23	Friction and wear	5
9	Relaxation	11	24	Finite element simulation	5
10	Elevated temperature	10	25	Galfan-coated steel cable	5
11	Finite element analysis	10	26	Experimental investigation	5
12	Fretting fatigue	10	27	Constitutive equation	5
13	Behavior	10	28	Bending stiffness	5
14	Stiffness	9	29	Steel strand	4
15	Fatigue	9	30	Finite element model	4

**Table 2:** Elastic modulus of cables.

Type		Elastic Modulus (MPa)
SPWS		(1.9~2.0) × 10^5^
Steel wire rope	Single-strand	1.4 × 10^5^
	Multi-strand	1.1 × 10^5^
SS	Galvanized	(1.85~1.95) × 10^5^
	High-strength low-relaxation	(1.85~1.95) × 10^5^
Steel rod		2.06 × 10^5^

**Table 3:** Experimental studies on cables’ mechanical properties under various temperature conditions.

Scholar	Specimen	Grade (MPa)	Treatment Conditions	Temp. Range (°C)
Zhou et al. [77]	SS	1860	Performed during exposure to high temperatures	20–700
Du et al. [78]	SS	1860	Performed during exposure to high temperatures	20–800
Liu et al. [9]	Steel wires	1860	Performed during exposure to high temperatures	20–700
	SS	1860	Performed during exposure to high temperatures	20–700
			Performed after exposure to high temperatures	100–1000
Liu et al. [79]	Steel wires	1860	Performed after exposure to high temperatures	15–1000
	SS	1860		1000
Lou et al. [11]	SS	1860	Performed during exposure to high temperatures	20–600
Sun et al.	GSS [80]	/	/	Room temperature
	GSS [12]	1670	Performed during exposure to high temperatures	30–600
	GSS [81]	1670	Performed after exposure to high temperatures	100–500
	SSC [13]	1500	Performed during exposure to high temperatures	30–600
	SSC [82]	1500	Performed after exposure to high temperatures	200–600
Wang et al. [83]	GSS LCR	1670 1570	Performed during exposure to high temperatures	20–700 20–700
Fontanari et al. [84]	Steel wires	/	Performed during exposure to high temperatures	100–600
	LCR	/	/	Room temperature

## Data Availability

No new data were created or analyzed in this study.
